# Enhancing Energy Efficiency in Telehealth Internet of Things Systems Through Fog and Cloud Computing Integration: Simulation Study

**DOI:** 10.2196/50175

**Published:** 2024-03-06

**Authors:** Yunyong Guo, Sudhakar Ganti, Yi Wu

**Affiliations:** 1 Computer Science Department, University of Victoria Victoria, BC Canada

**Keywords:** cloud computing, energy-efficient, fog computing, Internet of Things, IoT, telehealth

## Abstract

**Background:**

The increasing adoption of telehealth Internet of Things (IoT) devices in health care informatics has led to concerns about energy use and data processing efficiency.

**Objective:**

This paper introduces an innovative model that integrates telehealth IoT devices with a fog and cloud computing–based platform, aiming to enhance energy efficiency in telehealth IoT systems.

**Methods:**

The proposed model incorporates adaptive energy-saving strategies, localized fog nodes, and a hybrid cloud infrastructure. Simulation analyses were conducted to assess the model’s effectiveness in reducing energy consumption and enhancing data processing efficiency.

**Results:**

Simulation results demonstrated significant energy savings, with a 2% reduction in energy consumption achieved through adaptive energy-saving strategies. The sample size for the simulation was 10-40, providing statistical robustness to the findings.

**Conclusions:**

The proposed model successfully addresses energy and data processing challenges in telehealth IoT scenarios. By integrating fog computing for local processing and a hybrid cloud infrastructure, substantial energy savings are achieved. Ongoing research will focus on refining the energy conservation model and exploring additional functional enhancements for broader applicability in health care and industrial contexts.

## Introduction

### Overview

Health care is a critical global industry, and the advent of the Internet of Things (IoT) and cloud computing has significantly transformed health care system management. The ever-increasing data volume generated by these systems demands efficient, energy-saving computing platforms. In response, we present a groundbreaking energy-efficient model that seamlessly integrates telehealth IoT devices with fog and cloud computing–based platforms, offering a unique solution to address energy efficiency and data processing challenges. The rapid proliferation of IoT devices in health care has transformed approaches to patient care, diagnostics, and treatment. Telehealth, a key IoT health care application, has proven its potential to enhance care quality, reduce costs, and boost patient satisfaction. Despite these benefits, issues such as scalability, latency, and resource management persist, along with the significant challenge of energy consumption in smart devices within fog environments [1]. As a result, energy efficiency must be prioritized in the development of fog computing solutions, given its substantial impact on reducing carbon footprints and mitigating climate change effects. The large-scale deployment of telehealth IoT devices also raises concerns about energy consumption and data processing efficiency in delivering quality health care services. Intelligent choices for telehealth IoT devices should consider factors such as device movement or relevant environmental conditions to optimize energy consumption and manage associated equipment effectively. Typically, cloud-based analytical assessments are conducted for these devices [2]. To tackle these challenges, we propose an energy-saving model that integrates telehealth IoT devices with a fog and public or private cloud computing–based platform. The aim of the study is to develop an energy-efficient model that optimally integrates telehealth IoT devices with fog and cloud computing platforms, addressing challenges related to energy consumption, scalability, and data processing efficiency in delivering quality medical and patient services.

Telehealth IoT devices refer to a wide range of interconnected medical devices and sensors that facilitate remote health care services. These devices enable the continuous monitoring of patient’s vital signs, timely diagnostics, and personalized treatment plans, thereby improving the overall quality of health care. Some common examples of telehealth IoT devices include wearable health monitors, smart glucose meters, remote patient monitoring systems, and telemedicine platforms. The large-scale deployment of telehealth IoT devices presents several challenges [3], including energy consumption, data management, latency, security and privacy, scalability, and interoperability.

### Related Work

Telehealth has emerged as a promising solution to address various challenges in health care, such as accessibility, cost, and quality of care [4]. IoT devices play a significant role in telehealth applications, enabling remote monitoring, diagnostics, and treatment [2]. Several studies have investigated the implementation and efficacy of telehealth IoT devices in various health care scenarios, highlighting their potential to improve patient outcomes and satisfaction [5,6]. Fog computing has been identified as a promising approach to address the challenges associated with large-scale IoT deployments in health care, such as latency, energy consumption, and data management [7,8]. Researchers have proposed several fog computing–based architectures and frameworks for health care applications, demonstrating the potential of fog computing to enhance the performance and efficiency of telehealth IoT devices [9-11]. Cloud computing has gained significant attention in health care due to its scalability, cost-effectiveness, and advanced data analytics capabilities [12,13]. Several studies have explored the integration of cloud computing with telehealth IoT devices, showing its potential to address the challenges related to data storage, processing, and security [14-16].

Energy efficiency is critical in large-scale IoT deployments, especially in health care applications where device longevity and reliability are essential [17]. Researchers have proposed various energy-saving models and strategies for IoT devices, including adaptive power management [18], energy-efficient routing protocols [19], and data compression techniques [20]. However, few studies have specifically focused on energy-saving models that integrate telehealth IoT devices with fog and cloud computing–based platforms. The integration of fog and cloud computing has emerged as a promising approach to harness the benefits of both paradigms and address the challenges of large-scale IoT deployments [21,22]. Several studies have proposed models and frameworks that combine fog and cloud computing for various IoT applications [23-25], but few have specifically targeted energy-saving in telehealth IoT deployments.

In recent years, several simulation methods have been developed to study the integration of fog nodes in IoT devices and cloud computing. Gupta et al [26] introduced iFogSim, a toolkit for modeling and simulating resource management techniques in IoT, edge, and fog computing environments. Oueis et al [27] presented a simulation study on load distribution in small-cell cloud computing using fog computing and proposed a fog balancing technique to optimize resource allocation and reduce latency. Barcelo et al [28] explored IoT-cloud service optimization through simulation in smart environments, presenting a novel optimization framework that uses fog nodes to reduce latency and energy consumption. Zeng et al [29] conducted a comparative study of IoT cloud and fog computing simulations using iFogSim and Cooja, discussing the advantages and limitations of both simulators and providing insights into selecting an appropriate tool for specific scenarios. Lastly, Byers and Wetterwald [30] discussed the concept of fog computing and its importance in distributing data and intelligence for IoT resiliency and scalability, presenting various simulation models and techniques used to evaluate the performance of fog computing in IoT environments. Several studies have focused on the Yet Another Fog Simulator (YAFS) framework, a simulator designed for modeling and simulating fog computing environments in IoT scenarios. Bermejo et al [31] introduced YAFS, presenting the architecture, components, and use cases of the simulator, demonstrating its effectiveness in modeling and simulating fog computing deployments. García et al [32] showcased YAFS’s ability to model and simulate fog computing scenarios and analyze the performance of different scheduling algorithms. In a comparative study, Rodríguez et al [33] analyzed the features, capabilities, and limitations of YAFS, iFogSim, and EdgeCloudSim simulators, providing insights into selecting the most suitable tool for specific fog computing scenarios.

Several studies have explored different aspects of telehealth simulations, fog nodes, IoT devices, and cloud computing for energy-saving purposes. Aazam and Huh [34] discussed a smart gateway–based communication approach using fog computing for energy-saving in the Cloud of Things, which can be applied to various IoT applications, including telehealth. Verma and Sood [35] presented a fog-assisted IoT framework for patient health monitoring in smart homes, focusing on energy efficiency and reduced latency through a decentralized fog computing architecture. Koubaâ et al [36] proposed a fog-based emergency and health care system for smart cities, which leverages fog nodes and IoT devices to optimize energy consumption and provide real-time health care services, thus addressing energy-saving concerns in telehealth scenarios. Sareen et al [37] introduced an energy-efficient context-aware framework for managing application execution in cloud-fog environments, which can potentially improve energy efficiency in various IoT applications, including telehealth scenarios.

## Methods

### Model Overview

The proposed energy-saving model is designed to integrate telehealth IoT devices with a fog and cloud computing–based platform, leveraging the advantages of both paradigms to optimize energy consumption and ensure efficient data processing. The model comprises 3 main components: IoT devices, fog nodes, and public or private cloud servers, which are interconnected through a communication network.

**Figure 1:**
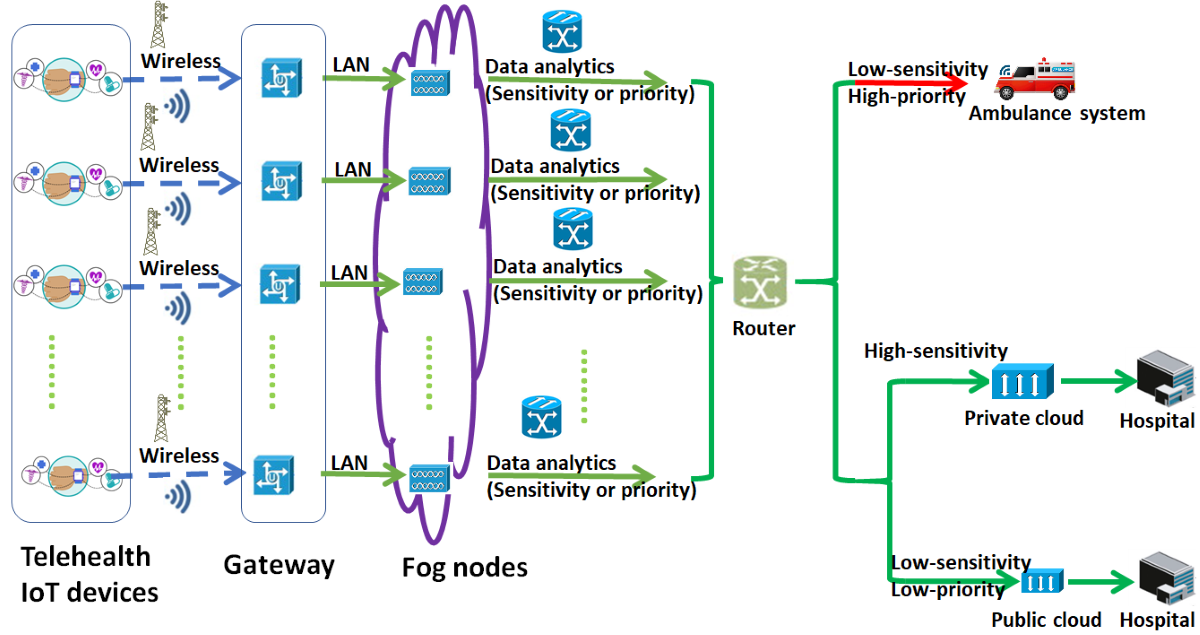
Telehealth Internet of Things (IoT) devices integrated with fog nodes and a private or public cloud architecture model. LAN: local area network.

The model architecture is shown in Figure 1 [38].

1. IoT devices: telehealth IoT devices, such as wearables, sensors, and remote monitoring systems, collect and transmit patient data in real time. These devices can dynamically adjust their power states (eg, active, idle, and sleep) based on their tasks, reducing energy consumption without compromising the quality of health care services.
2. Fog nodes: fog nodes, located near IoT devices, serve as intermediate processing units. They perform localized data processing, analytics, and storage, reducing the amount of data transmitted to the cloud servers.
3. Cloud servers: cloud servers provide a robust infrastructure for large-scale data storage, processing, and advanced analytics.
4. Communication network: a communication network connects IoT devices, fog nodes, and cloud servers, enabling seamless data transmission and task allocation.

The telehealth IoT network depicted in the diagram is designed to ensure efficient and secure data transmission between the different network components. To ensure network security, firewalls are placed between IoT devices and fog nodes. This ensures that unauthorized access to the network is prevented, and sensitive health care data are kept confidential. To process the data requests, the fog nodes are equipped with data analytics functions that enable them to intelligently assign different types of requests to either fog nodes, a private cloud, or a public cloud. This intelligent decision-making process is more effective and efficient than the traditional “first-come, first-served” approach. The gateway and router are integral components in the network that enable seamless data transmission between the fog nodes and cloud instances. The gateway acts as the entry point for the network and connects the IoT devices to the local fog nodes. It is responsible for handling the data transmission and conversion between different protocols used by IoT devices and fog nodes. The router, on the other hand, is responsible for directing the data traffic between the fog nodes and cloud instances based on various factors, such as the sensitivity, priority, and latency requirements of the data. It determines which data should be sent to the cloud and which data should be processed by the fog nodes, ensuring efficient use of network resources. The router also handles the communication between different fog nodes and cloud instances, enabling seamless data transmission across the network.

**Figure 2:**
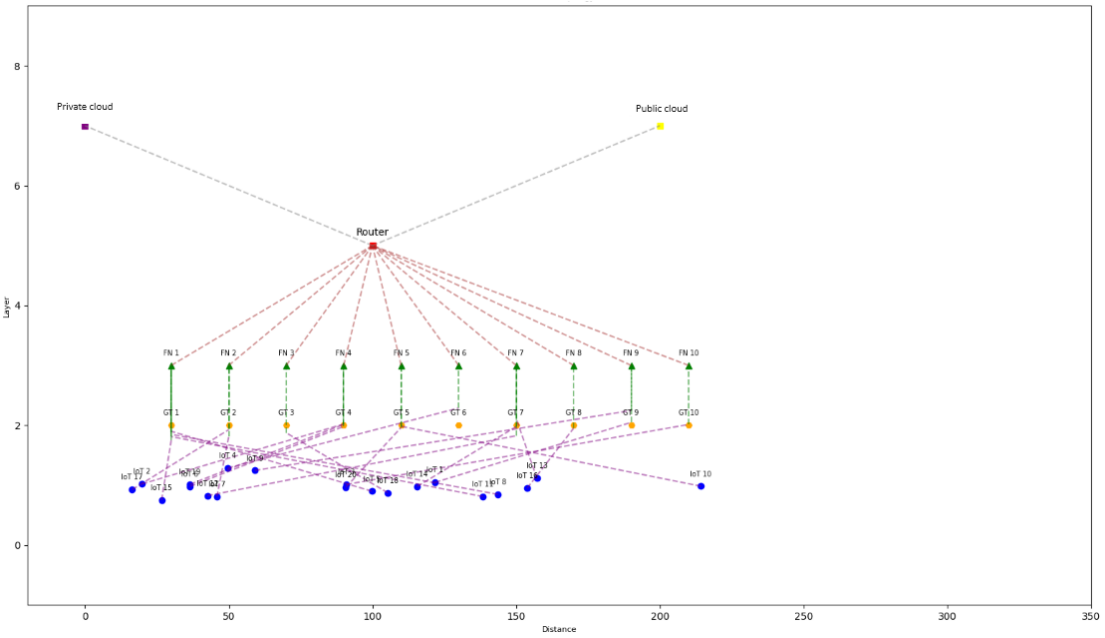
Network topology for the proposed Internet of Things (IoT) devices integrated with fog nodes and cloud. A brief overview of the components in the network topology: (1) IoT devices (blue circles) represent individual IoT devices in the network, each associated with a specific fog node. (2) Gateways (GT; orange hexagons) are used to connect the IoT devices to the fog nodes. (3) Fog nodes (FN; green triangles) are intermediate computing resources that process and store data from IoT devices. (4) A router (red square) connects the fog nodes to the private cloud and public cloud. (5) A private cloud (purple square) and a public cloud (yellow square) are the 2 cloud resources in the network.

The proposed telehealth IoT system shown in Figure 2 intelligently manages data transmission based on the sensitivity and priority of the data. For high-sensitivity data, the system ensures privacy and security by sending it directly to the private cloud, which then transfers the data to authorized health facilities as needed. On the other hand, low-sensitivity but high-priority requests are routed to the fog nodes as they have the capability to process urgent requests in a timely manner, such as in life-threatening emergency situations. These requests are then transmitted to ambulance systems for immediate treatment. Lastly, data with low sensitivity and low priority are sent to the public cloud as it has more space and scalability to store and process such data. The public cloud can also serve as a repository for future research or clinical purposes.

By allocating data transmission to the appropriate destination, the proposed system ensures efficient and effective data processing while maintaining privacy and security for sensitive health care data. This approach also optimizes energy consumption and reduces latency, ensuring a seamless experience for health care providers and patients. The categorization of high and low sensitivity and high and low priority data sent from telehealth IoT monitor devices can depend on various factors, including the specific use case, regulatory requirements, and patient needs. One possible approach could be to use threshold values based on vital signs such as pulse and heartbeat to categorize the data. For example, data related to vital signs that fall within normal ranges may be classified as low sensitivity and low priority, as they do not require immediate attention. Data related to vital signs that are outside the normal range but do not pose an immediate threat to the patient’s health may be classified as low sensitivity but high priority. Data related to vital signs that indicate a life-threatening condition, such as cardiac arrest, may be classified as high sensitivity and high priority, requiring immediate attention from health care providers.

The exact vital sign thresholds for patient emergencies can vary depending on a range of factors, including the age and health condition of the patient, the specific symptoms, and other medical history [39]. In general, some common vital sign thresholds used to classify emergencies include the following:

- Heart rate: a heart rate above 100 bpm or below 60 bpm may be indicative of an emergency [40].
- Blood pressure: a systolic blood pressure (the top number) above 180 mm Hg or below 90 mm Hg, or a diastolic blood pressure (the bottom number) above 110 mm Hg or below 60 mm Hg may indicate an emergency [41].
- Respiratory rate: a respiratory rate above 30 breaths per minute or below 10 breaths per minute may be indicative of an emergency [42].
- Oxygen saturation: an oxygen saturation level below 90% may be indicative of an emergency [43].

However, it is important to note that this is just one possible approach, and the categorization of data should be customized based on the specific needs of the patient and health care provider. It is also important to comply with relevant regulations and ensure patient privacy and security while handling sensitive health care data.

### Key Components and Energy-Saving Strategies

The proposed energy-saving model incorporates several strategies to minimize energy consumption.

#### Task Allocation

The model intelligently allocates tasks between fog nodes and cloud servers based on factors such as computational capacity, proximity to IoT devices, and current workload. This ensures efficient data processing and reduces energy consumption for data transmission.

#### Adaptive Power Management

IoT devices and fog nodes can dynamically adjust their power states (eg, active, idle, and sleep) based on their tasks and workload, ensuring optimal energy consumption without compromising the quality of health care services.

#### Data Compression and Aggregation

Data generated by IoT devices can be compressed and aggregated at the fog nodes before transmission to cloud servers, reducing the volume of data transmitted and, consequently, energy consumption.

#### Network Optimization

The communication network can be optimized to minimize energy consumption by using energy-efficient routing protocols and minimizing transmission distances.

### Simulation Study

To assess the effectiveness of the proposed energy-efficient model, we developed a simulation model that emulates a real-world telehealth scenario focused on remote patient monitoring. Within this simulated scenario, numerous patients with chronic conditions are equipped with wearable IoT devices that continuously track vital signs such as heart rate, blood pressure, and blood glucose levels. The gathered data are processed and analyzed by the integrated fog and cloud computing–based platform, facilitating timely diagnostics and personalized treatment plans. Textbox 1 contains the pseudocode for the provided code.

In short, this code is devised to emulate an IoT network, scrutinizing the influence of fog nodes on energy consumption while providing a graphical representation of the network architecture to elucidate the connections among IoT devices, fog nodes, and cloud services. IoT devices transmit data to their corresponding destinations, such as fog nodes, private clouds, or public clouds, contingent upon their sensitivity and priority attributes. The energy expenditure for data transmission to these target locations differs; hence, the code performs a simulation to determine the residual energy for each device under 2 distinct scenarios (ie, with and without fog nodes). Subsequently, the code generates a bar chart to depict the energy consumption patterns of IoT devices in both cases, and it stores the energy usage outcomes in 2 separate Microsoft Excel (Microsoft Corporation) files, enabling in-depth examination and assessment of the results.

The algorithm of the code can be analyzed in the following steps:

1. Initialization: create IoT devices, fog nodes, and cloud instances with their respective properties.
2. Connection: connect IoT devices to fog nodes and then fog nodes, and determine which data are transferred to cloud instances (private and public). Each device is connected to a corresponding fog node.
3. Data transmission simulation: simulate data transmission from IoT devices to their respective fog nodes, and then fog nodes assign the requests to a private cloud or a public cloud based on their priority and sensitivity. If the sensitivity of the device is “high,” data are sent to the private cloud. If the sensitivity is “low” and the priority is “high,” there is a chance (defined by *self.fog_node [chance]*) that data are sent to the fog node. If this condition is not met, the device does not send data. If the sensitivity is “low” and the priority is “low,” data are sent to the public cloud.
4. Energy consumption calculation: calculate the energy consumed by each IoT device during data transmission, considering the parameter of latency. Different energy costs are associated with sending data to different destinations (fog nodes, private cloud, or public cloud).
5. Comparison: compare the energy consumption of IoT devices when using fog nodes and when not using fog nodes. (1) Run the simulation with fog nodes connected and store the remaining energy for each device. (2) Reset the energy of the devices, disconnect them from fog nodes, and run the simulation without fog nodes, storing the remaining energy for each device again.
6. Export the energy usage results to Excel files for both cases (with and without fog nodes).
7. Visualize the network topology with devices, fog nodes, and clouds using the *show_topology* function.

In this enhanced task allocation algorithm, we incorporate additional factors such as device distance, data sensitivity, request priority, energy consumption, and latency to provide a more sophisticated and adaptable solution for large-scale telehealth IoT deployments. The algorithm starts by defining parameters such as latency, distance, energy consumption, and sensitivity thresholds. The task queues for each fog node and cloud server are initialized. For each task type, average processing times, energy consumption, sensitivity, and priority are calculated for each fog node and cloud server according to some random data sent from each IoT device. The algorithm then assesses the latency, priority, sensitivity, and energy consumption for transmitting data from each device to each fog node and then to the private and public cloud server. Based on these factors, the algorithm selects the optimal fog node and cloud server for each device, ensuring that the chosen nodes meet the specified thresholds for latency, sensitivity, and energy consumption. Tasks are allocated to fog nodes and cloud servers based on data sensitivity, priority, and energy consumption, ensuring that the selected nodes do not exceed the energy consumption threshold. If no suitable nodes are found, alternative energy-saving strategies may be considered, or the energy consumption threshold may be adjusted. Finally, the tasks are processed in fog nodes and cloud servers based on their queues. By considering these additional factors, the enhanced algorithm can provide better energy-saving performance and adaptability to various telehealth scenarios, ensuring that the large-scale deployment of telehealth IoT devices on a fog and cloud computing–based platform remains efficient and effective.

Textbox 2 contains a task allocation algorithm for telehealth IoT devices integrated with a fog and cloud computing–based platform.

**The pseudocode for the provided code.**

**1. Define *IoTDevice* class**

- Initialize with attributes: *id*, *distance*, *priority*, *sensitivity*, *fog_node*, *private_cloud*, *public_cloud*, *energy*, *transmit_power*, *idle_power*, and *transmit_time*
- Define *send_data* method
  Check if the device has energy left
  Send high-sensitivity data to private cloud if sensitivity is high
  Send low-sensitivity, high-priority data to fog node if priority is high and *fog_node* exists
  Send low-sensitivity, low-priority data to public cloud otherwise
- Define *idle* method to reduce energy based on idle power and time

**2. Define *FogNode* class**

- Initialize with attributes: *id*, *public_cloud*, *energy*, *latency*, *devices*, *fog_energy_cost*, *cloud_energy_cost*, *chance*, *process_power*, *idle_power*, and *process_time*
- Define *connect_device* method to connect a device to the fog node
- Define *store_data* method to store data from a device with given sensitivity and priority
- Define *idle* method to reduce energy based on idle power and time
- Define *send_data* method to send data from connected devices based on their sensitivity and priority

**3. Define *PublicCloud* class**

- Initialize with attributes: *id*, *energy*, *latency*, and *cloud_energy_cost*
- Define *store_data* method to store data from a device

**4. Define *simulate* function**

- Create Internet of Things (IoT) devices with random priority and sensitivity
- Create fog nodes connected to a public cloud
- Connect IoT devices to fog nodes
- Connect IoT devices to private and public clouds
- Initialize lists to store energy usage results
- Simulate data transmission with fog nodes, store energy usage results
- Store energy usage with fog nodes
- Reset device energy
- Disconnect devices from fog nodes
- Simulate data transmission without fog nodes, store energy usage results
- Store energy usage without fog nodes
- Create energy usage bar plot and save as an image
- Save energy usage results to Microsoft Excel files (with and without fog nodes)

**Task allocation algorithm for telehealth Internet of Things devices integrated with a fog and cloud computing–based platform.**

**1. Define parameters**

- Internet of Things (IoT) devices: *D*= {*d1*, *d2*, ..., *dn*}
- Fog nodes: *F*= {*f*1, *f*2, ..., *f*m}
- Cloud servers: *C*= {*c*1, *c*2}
- Task types: *T*= {*t*1, *t*2, ..., *t*q}
- Data sensitivity threshold: *S*_*t*
- Data priority threshold: *P*r_t
- Latency threshold: *L*_*t*
- Energy consumption threshold: *E*_*t*

**2. Initialize task queues for each IoT device, fog node, and cloud server**

- *Q*_*D* [*i*] = {} for all *i* in *D*
- *Q*_*F* [*j*] = {} for all *j* in *F*
- *Q*_*C* [*l*] = {} for all *l* in *C*

**3. For each task type *t* in *T***

- Calculate the average processing time *P*_*t* and energy consumption *E*_*t* for each IoT device *i* in *D* and fog node *j* in *F*.
- Calculate average energy consumption *E*_*t*, sensitivity *S*_*t*, and priority *Pr_t* for each IoT device *i* in *D* and fog node *j* in *F*.

**4. For each device *d* in *D* and task type *t* in *T***

- Calculate the latency *L*_*dt* for transmitting data from device *d* to each fog node *i* in *F* and cloud server *j* in *C*.
- Calculate the priority *Pr_dt*, sensitivity *S*_*dt*, energy consumption *E*_*dt* for device *d*, and each fog node *i* in *F* and cloud server *j* in *C*.
- Find the fog node j* and cloud server l* with the minimum latency for device i*, considering *Pr_t*, *S_t*, and *E_dt*:
  j* = *argmin_j*(*L_dt*) for *j* in *F*, such that *L_dt* <= *L_t*, *Pr_dt* <= *Pr_t* and *S_dt* <= *S_t*
  l* = *argmin_l*(*L_dt*) for *l* in *C*, such that *L_dt* <= *L_t*, *Pr_dt* <= *Pr_t* and *S_dt* <= *S_t*

**5. Allocate tasks from devices to fog nodes and cloud servers: for each device *d* in *D* and task type *t* in *T***

- If *S*_*dt* [j*] ≤ *S*_*t*, then allocate task *t* to cloud server l* and add it to the queue: *Q_C* [l*].append((*d*, *t*))
- If *Pr_dt* [j*] ≤ *Pr_t*, then allocate task *t* to fog node j* and add it to the queue: *Q_F* [j*].append((*d*, *t*))
- Else if *Pr_dt* [l*] ≤ *Pr_t*, then allocate task *t* to cloud server l* and add it to the queue: *Q_C* [l*].append((*d*, *t*))
- Otherwise, consider alternative energy-saving strategies or adjust the energy consumption threshold *E*_*t*.

**6. Process tasks in fog nodes and cloud servers based on their queues**

- For each fog node *j* in *F*, process tasks in *Q_F* [*j*]
- For each cloud server *l* in *C*, process tasks in *Q_C* [*l*]

This algorithm aims to balance the load between fog nodes and cloud servers while considering latency, sensitivity, request priority, and energy consumption constraints. It can be further optimized by incorporating additional factors, such as device mobility. It is mainly focused on simulating data transmission from IoT devices to different destinations based on their priority and sensitivity, as well as comparing the energy consumption given the various latency when using fog nodes versus not using them. The objective is to demonstrate the potential benefits of using fog nodes in terms of energy efficiency for IoT devices.

## Results

### Parameters in Results

Based on the simulation results, we can analyze the impact of different parameters on the energy efficiency and performance of the proposed telehealth model with and without fog computing. The parameters in the results are given below.

#### Snapshot Interval

The *snapshot interval* parameter represents the frequency at which the IoT devices send their data to the fog nodes or cloud servers. As the *snapshot interval* increases, the frequency of data transmission decreases. With a *snapshot interval* of 1, the IoT devices are sending data continuously. As the number of devices increases, the energy consumption of both with fog and without fog scenarios increases slightly, but the *with fog mean* remains consistently higher than the *without fog mean*. With a *snapshot interval* of 5, the IoT devices are sending data less frequently, which results in reduced energy consumption. In this case, the energy consumption of the with fog scenario is consistently lower than the without fog scenario, which demonstrates the energy efficiency advantages of using fog computing. With a *snapshot interval* of 10, the IoT devices send data even less frequently, and the difference in energy consumption between the with fog and without fog scenarios becomes more pronounced. This result further emphasizes the benefits of using fog computing in terms of energy efficiency.

#### Number of Devices

The *number of devices* parameter refers to the number of telehealth IoT devices in the network. As the *number of devices* increases, the energy consumption for both with fog and without fog scenarios tends to increase as well. This is expected, as more devices lead to higher data transmission and processing loads. However, the increase in energy consumption is consistently smaller in the with fog scenario compared to the without fog scenario across all snapshot intervals. This shows that the proposed fog-based model is more scalable and can better handle the energy requirements of a growing number of devices.

#### With Fog Mean and Without Fog Mean

The *with fog mean* and *without fog mean* parameters represent the average energy consumption in the scenarios with and without fog computing, respectively. Across all snapshot intervals and several devices, the *with fog mean* is generally lower than the *without fog mean*, indicating that the fog-based model is more energy-efficient than the cloud-only model.

#### With Fog SD and Without Fog SD

The *with fog SD* and *without fog SD* parameters represent the SD of the energy consumption in the scenarios with and without fog computing, respectively. In general, the SD values are lower in the with fog scenario compared to the without fog scenario. This suggests that the energy consumption is more consistent and less variable in the fog-based model, which could lead to more predictable and stable system performance.

#### With Fog 95% CI and Without Fog 95% CI

The CI in the simulation code is a range within which a certain percentage of the population parameter is expected to lie, with a specified level of confidence. In the context of the provided simulation results, the 95% CIs represent the range within which the true mean performance of the system (either with or without fog computing) is likely to fall, with a certain level of confidence, typically 95%.

A 95% CI is calculated using the sample mean, sample SD, and sample size. The formula for a 95% CI is:

CI = sample mean ± (1.96 × [sample SD/sqrt {sample size}])

The 95% CI helps to quantify the uncertainty in the estimation of the true mean performance. A narrower 95% CI indicates a more precise estimate, while a wider interval suggests more uncertainty.

### Analysis of Results

Table 1 contains the summary of statistical results.

**Table 1:** Summary of statistical results.

Snapshot interval	Number of devices	With fog, mean (SD)	With fog, 95% CI	Without fog, mean (SD)	Without fog, 95% CI
1	10	90.43 (0.45)	90.11-90.76	89.74 (0.05)	89.69-89.79
1	20	90.53 (0.33)	90.30-90.77	89.74 (0.06)	89.69-89.79
1	30	90.61 (0.23)	90.45-90.78	89.74 (0.04)	89.71-89.78
1	40	90.55 (0.24)	90.38-90.72	89.76 (0.05)	89.71-89.90
5	10	87.39 (0.70)	86.89-87.89	86.04 (0.13)	85.94-86.13
5	20	86.59 (0.21)	86.44-86.73	85.91 (0.06)	85.86-85.95
5	30	87.02 (0.46)	86.70-87.34	86.01 (0.09)	85.95-86.08
5	40	87.30 (0.27)	87.12-87.50	86.00 (0.07)	85.95-86.05
10	10	82.85 (0.73)	82.34-83.38	81.36 (0.11)	81.27-81.44
10	20	83.28 (0.63)	82.83-83.72	81.42 (0.11)	81.33-81.50
10	30	82.62 (0.59)	82.80-83.03	81.33 (0.12)	81.24-81.43
10	40	82.7 (0.37)	82.43-82.95	81.36 (0.07)	81.31-81.41

Here is a step-by-step analysis of the results (Table 1):

1. Observe the “With fog, mean (SD)” and “Without fog, mean (SD)” columns for each combination of “Snapshot interval” and “Number of devices.” In all cases, the *with fog mean* is higher than the *without fog mean*, indicating that, on average, the remaining energy is higher when using fog computing.
2. Look at the 95% CIs for both “with fog” and “without fog” scenarios. If the 95% CIs do not overlap, it suggests that the difference in energy remaining between the 2 scenarios is statistically significant. For example, in the first row (snapshot interval: 1, number of devices: 10), the “with fog, 95% CI” is 87.98-89.45, and the “without fog, 95% CI” is 84.90-87.47. Since these intervals do not overlap, there is strong evidence that using fog computing leads to significantly higher energy remaining for this specific combination of parameters.
3. Compare the width of the 95% CIs for each scenario. A narrower 95% CI indicates a more precise estimate of the true population mean. For most 95% CI values, the “with fog, 95% CI” is narrower than the “without fog, 95% CI,” suggesting that the “with fog” scenario has a more precise estimate.
4. Analyze the trends as the number of devices increases within each snapshot interval. In general, the energy remaining in both scenarios decreases as the number of devices increases. However, the rate of decrease seems to be lower when using fog computing.
5. Observe the trends as the snapshot interval increases for each group of devices. As the snapshot interval increases, the energy remaining for both scenarios decreases, suggesting that less frequent snapshots may lead to less energy conservation. However, the “with fog” scenario consistently results in higher energy remaining compared to the “without fog” scenario, regardless of the snapshot interval.

In conclusion, based on the analysis of the means and 95% CIs, it appears that using fog computing is beneficial for conserving energy, especially when the number of devices and the snapshot intervals increase. The difference in energy remaining is statistically significant in most cases, and the “with fog” scenario consistently outperforms the “without fog” scenario.

Therefore, the simulation results demonstrate that the proposed fog-based telehealth model provides improved energy efficiency and scalability compared to a cloud-only model, especially when the IoT devices send data less frequently. The lower energy consumption and SD values in the with fog scenario indicate that fog computing is a viable solution for managing energy requirements and maintaining consistent performance in telehealth IoT networks. Furthermore, we conducted the sensitivity simulation analysis to systematically investigate the impact of variations in model parameters on the simulation outcomes. Sensitivity analysis helps in understanding how different input parameters influence the system’s behavior and performance and identifies critical factors that have a significant effect on the results. According to the simulation code running, the sensitivity analysis was performed for various parameters such as *transmit_power*, *idle_power*, *latency*, and *energy_cost*. By varying these parameters across a range of values, the impact on the energy remaining in IoT devices with and without fog nodes can be evaluated.

Table 2 compares the mean energy remaining for IoT devices with and without fog nodes for each energy cost value. The “Mean difference” column shows the difference in mean energy remaining, with positive values indicating that devices with fog nodes have higher energy remaining compared to those without fog nodes. In the with fog scenario, the mean energy remaining for devices with fog nodes stays relatively stable, ranging from a minimum of 93 to a maximum of 95 across different energy costs. In the without fog scenario, the mean energy remaining for devices without fog nodes also remains relatively stable, ranging from a minimum of 91 to a maximum of 92 across different energy costs.

**Table 2:** Sensitivity analysis with energy cost.

Energy cost	With fog, mean (SD)	Without fog, mean (SD)	Mean difference
0.20	94 (5)	91 (4)	1.72
0.26	93 (6)	92 (5)	0.33
0.32	94 (4)	92 (7)	1.72
0.38	93 (5)	91 (2)	1.72
0.44	94 (3)	91 (5)	2.75
0.5	93 (2)	91 (4)	1.71
0.56	95 (2)	92 (2)	1.72
0.62	94 (3)	92 (4)	1.37
0.68	93 (2)	92 (2)	0.68
0.74	94 (4)	92 (3)	1.02
0.80	95 (6)	92 (3)	2.06

Based on the sensitivity analysis of energy cost, the mean energy remaining for IoT devices with fog nodes is consistently higher than that of devices without fog nodes across all energy cost values. This indicates that IoT devices with fog nodes perform better in terms of energy consumption as compared to devices without fog nodes.

Table 3 compares the mean energy remaining for IoT devices with and without fog nodes for each latency parameter value. The “Mean difference” column shows the difference in mean energy remaining, with positive values indicating that devices with fog nodes have higher energy remaining compared to those without fog nodes. In the with fog scenario, the mean energy remaining for devices with fog nodes stays relatively stable, ranging from a minimum of 94 to a maximum of 95 across different latency values. In the without fog scenario, the mean energy remaining for devices without fog nodes also remains relatively stable, ranging from a minimum of 91 to a maximum of 93 across different latency values.

Based on the sensitivity analysis of latency, the mean energy remaining for IoT devices with fog nodes is consistently higher than that of devices without fog nodes across all latency parameter values. This indicates that IoT devices with fog nodes perform better in terms of energy consumption as compared to devices without fog nodes.

Table 4 compares the mean energy remaining for IoT devices with and without fog nodes for each idle power value. The “Mean difference” column shows the difference in mean energy remaining, with positive values indicating that devices with fog nodes have higher energy remaining compared to those without fog nodes. In the with fog scenario, the mean energy remaining for devices with fog nodes stays relatively stable, ranging from a minimum of 93 to a maximum of 95 across different idle power values. In the without fog scenario, the mean energy remaining for devices without fog nodes also remains relatively stable, ranging from a minimum of 90 to a maximum of 92 across different idle power values.

**Table 3:** Sensitivity analysis with latency.

Latency parameter	With fog, mean (SD)	Without fog, mean (SD)	Mean difference
0.20	94 (4)	92 (4)	2.06
0.26	93 (3)	91 (4)	2.06
0.32	94 (2)	93 (1)	1.72
0.38	95 (2)	92 (2)	2.06
0.44	94 (2)	92 (2)	1.72
0.5	94 (3)	91 (3)	2.4
0.56	94 (4)	92 (5)	2.06
0.62	94 (2)	92 (2)	2.06
0.68	94 (3)	92 (3)	1.71
0.74	94 (5)	93 (3)	0.68
0.80	94 (2)	92 (4)	0.68

**Table 4:** Sensitivity analysis with idle power.

Idle power	With fog, mean (SD)	Without fog, mean (SD)	Mean difference
0.5	95 (2)	92 (2)	2.06
0.6	94 (2)	92 (4)	1.71
0.7	95 (3)	92 (4)	2.41
0.8	93 (2)	90 (3)	1.72
0.9	94 (4)	92 (4)	1.03
1.0	93 (5)	91 (4)	1.02
1.1	95 (4)	92 (6)	1.71
1.2	94 (4)	91 (5)	2.06
1.3	95 (2)	91 (2)	2.75
1.4	94 (1)	91 (4)	2.06
1.5	94 (2)	92 (4)	1.72

Based on the sensitivity analysis of idle power, the mean energy remaining for IoT devices with fog nodes is consistently higher than that of devices without fog nodes across all idle power values. This indicates that IoT devices with fog nodes perform better in terms of energy consumption as compared to devices without fog nodes.

Table 5 compares the mean energy remaining for IoT devices with and without fog nodes for each transmit power value. The “Mean difference” column shows the difference in mean energy remaining, with positive values indicating that devices with fog nodes have higher energy remaining compared to those without fog nodes. In the with fog scenario, the mean energy remaining for devices with fog nodes stays relatively stable, ranging from a minimum of 94 to a maximum of 96 across different transmit power values. In the without fog scenario, the mean energy remaining for devices without fog nodes also remains relatively stable, ranging from a minimum of 91 to a maximum of 92 across different transmit power values.

**Table 5:** Sensitivity analysis with transmit power.

Transmit power	With fog, mean (SD)	Without fog, mean (SD)	Mean difference
0.5	94 (3)	91 (2)	1.37
0.6	95 (4)	92 (3)	2.41
0.7	94 (2)	92 (4)	1.37
0.8	94 (3)	91 (5)	1.71
0.9	94 (2)	92 (4)	1.37
1.0	94 (5)	91 (2)	2.06
1.1	94 (6)	92 (3)	1.37
1.2	94 (6)	92 (3)	1.02
1.3	95 (4)	92 (3)	2.40
1.4	96 (2)	91 (2)	3.79
1.5	95 (2)	91 (1)	3.44

Based on the sensitivity analysis of transmit power, the mean energy remaining for IoT devices with fog nodes is consistently higher than that of devices without fog nodes across all transmit power values. This indicates that IoT devices with fog nodes perform better in terms of energy consumption as compared to devices without fog nodes.

### Ethical Considerations

The study did not apply for any ethical approval, as the research did not involve any human participants or animals [44].

## Discussion

### Overview

The simulation study results indicate that the proposed energy-saving model could be effective in reducing energy consumption in real-world telehealth scenarios. Key findings include the following:

1. Scalability: the model demonstrates the ability to accommodate an increasing number of IoT devices without compromising performance, energy efficiency, or quality of health care services.
2. Task allocation algorithm: the proposed task allocation algorithm outperforms other algorithms in terms of energy efficiency and data processing efficiency, indicating its effectiveness in balancing the workload between fog nodes and cloud servers.
3. Energy consumption metrics: the overall energy consumption is reduced across all levels, demonstrating the success of the model’s energy-saving strategies, such as adaptive power management, data compression, and network optimization.

The code and methodology described aim to simulate an IoT network with different components (IoT devices, fog nodes, and cloud servers) and analyze the impact of fog nodes on energy consumption. The code creates and connects these components and simulates data transmission, storage, and energy consumption for IoT devices, fog nodes, and cloud servers. The simulation results are analyzed to understand the network behavior and demonstrate the potential benefits of using fog nodes for energy efficiency.

Our novel energy-efficient model integrates fog and cloud computing paradigms to optimize data processing for telehealth IoT devices without compromising real-time health care services. This stands out from previous works by enabling localized data processing through the incorporation of fog computing. This intermediary layer, situated between IoT devices and cloud servers, effectively reduces latency and data transfer overhead. The concurrent use of public and private cloud computing further fortifies the system’s infrastructure, allowing for the handling of large data volumes and resource-intensive computations. The model enables localized data processing by incorporating fog computing as an intermediary layer between IoT devices and public or private cloud servers, effectively reducing latency and data transfer overhead. Simultaneously, public and private cloud computing provides a robust infrastructure for handling large data volumes and performing resource-intensive computations. The primary goal of this model is to minimize energy consumption through intelligent task allocation between fog nodes and cloud servers, by considering their computational capacity and proximity to IoT devices. This task allocation process also considers various sensitivity and priority levels within the health care context, ensuring prompt responses to critical and high-sensitivity requests. Our innovative model strategically integrates fog and cloud computing, aiming to establish an energy-efficient telehealth IoT system capable of adeptly managing data processing and delivering real-time health care services, accommodating various levels of sensitivity and priorities. While these aspirations suggest promising opportunities for further optimization and diverse applications within health care contexts, it is crucial to note that the subsequent simulation method serves to objectively assess the model’s effectiveness and efficiency. The empirical evidence derived from the simulation provides a foundation for a more nuanced understanding of the model’s capabilities and potential benefits. This is because exploring diverse large-scale network topologies is rarely feasible in the real world. Although the requirements for such a simulator are straightforward—providing a detailed, accurate, and granular model of all components—implementing corresponding simulators demands considerable effort.

The primary strength of our model lies in its holistic approach toward minimizing energy consumption. The intelligent task allocation mechanism, considering computational capacity and proximity to IoT devices, ensures a fine balance. Furthermore, the incorporation of sensitivity and priority levels within the health care context enhances the model’s responsiveness to critical requests. The synergistic integration of fog and cloud computing contributes to the creation of an energy-efficient telehealth IoT system capable of real-time data processing in accordance with varying sensitivity levels and priorities.

Despite the positive outcomes, several limitations should be acknowledged. (1) Simulation environment realism: the simulation, while essential for its controlled environment, may not perfectly mirror real-world complexities. Variations in network behaviors and external factors may influence results differently in practical implementations. (2) Sensitivity analysis scope: the sensitivity analysis, while comprehensive, focused on specific parameters such as energy cost, latency, idle power, and transmit power. Additional parameters and their potential interactions may provide a more nuanced understanding of the model’s behavior. (3) Simplifications in simulation: certain simplifications, inherent in simulation models, may oversimplify the intricacies of a live telehealth IoT deployment. Real-world complexities such as device failures, communication errors, or dynamic changes in the environment are challenging to fully capture.

To address these limitations and advance the research, the following suggestions should be considered. (1) Future studies should aim for more realistic simulation environments, incorporating dynamic factors and diverse network topologies to enhance the model’s external validity. (2) Expanding the scope of sensitivity analysis to include a broader range of parameters and exploring their interactions could provide a more comprehensive understanding of the model’s performance under diverse conditions. (3) The development of more sophisticated simulators, despite their challenges, remains crucial. Detailed, accurate, and granular models of all components can better simulate the intricacies of large-scale IoT-fog-cloud systems.

While our model exhibits significant promise in reducing energy consumption and enhancing data processing efficiency in telehealth IoT scenarios, ongoing refinement and exploration of diverse scenarios will contribute to its continued evolution and real-world applicability.

### Conclusion

This paper provides a compelling model for the use of fog and cloud computing–based platforms in telehealth IoT deployments to reduce energy consumption, improve data processing efficiency, and maintain high-quality health care services. The model leverages the strengths of both fog and cloud computing paradigms to address the challenges associated with large-scale telehealth IoT deployments, such as energy consumption, data processing efficiency, latency, security, and privacy. The simulation results show that the proposed fog-based model significantly reduces energy consumption compared to the cloud-only model while maintaining high-quality data processing and transmission. Moreover, the methodology described in this paper provides a comprehensive approach to analyzing network performance and energy consumption, which includes examining the impact of various parameters, such as the number of devices, fog node deployment, task allocation algorithm, energy consumption metrics, and performance metrics. Sensitivity analyses were conducted with respect to energy cost, latency, idle power, and transmit power, consistently showing that IoT devices with fog nodes had higher mean energy remaining compared to devices without fog nodes. This approach allows for a more detailed understanding of the network behavior and potential bottlenecks and provides insights into how to optimize the model to be more resilient and efficient. The simulation results and methodology demonstrate the effectiveness of the proposed model and provide a roadmap for future research in this area. We demonstrated the effectiveness of the proposed model in reducing energy consumption while, more importantly, ensuring efficient data processing and maintaining the quality of health care services. The proposed model can help health care providers and stakeholders improve patient care and outcomes while reducing costs and energy consumption.

## Abbreviations

IoT: Internet of Things
YAFS: Yet Another Fog Simulator
